# Biomechanical evaluation of reconstruction of the posterior complex in restorative laminoplasty with miniplates

**DOI:** 10.1186/s12891-023-06380-3

**Published:** 2023-04-14

**Authors:** Jianmin Chen, Guoyin Liu, Tianyi Bao, Yuansheng Xu, Hu Luo, Yu Wu, Dawei Cai, Feng Qin, Jianning Zhao

**Affiliations:** 1 Department of Orthopaedics, Jinling Hospital of Nanjing Medical University, 305 Zhongshan East Road, Nanjing, 210000 Jiangsu Province China; 2 Faculty of Engineering and the Environment, University of Southampton, Southampton, UK

**Keywords:** Biomechanics, Laminoplasty, Laminectomy, Miniplates, Screws

## Abstract

**Objective:**

To evaluate the biomechanical effects of different miniplates on restorative laminoplasty.

**Methods:**

Assembled restorative laminoplasty models were developed based on 3D printed L4 lamina. Based on different internal fixations, the research was divided into H-shaped miniplates (HSMs) group, two-hole miniplates (THMs) group, and L-shaped miniplates (LSMs) group. The static and dynamic compression tests were analyzed to investigate the biomechanical effects of different internal fixations in restorative laminoplasty, until the failure and fracture of miniplates, or the collapse of miniplates. The static compression tests adopted the speed control mode, and the dynamic fatigue compression tests adopted the load control mode.

**Results:**

The “door close” and the collapse of lamina occurred in THMs group and LSMs group, and plate break occurred in LSMs group. However, these phenomenon was absent in HSMs group, and only plate crack around a screw and looseness of a screw tail cap were found in HSMs group. The sustainable yield load of HSMs group was greater than that of THMs group and LSMs group (P < 0.05). No significant difference in yielding-displacement was found between HSMs group and LSMs group (P > 0.05), while both were much less than that of THMs (P < 0.05). Moreover, the compressive stiffness and the axial displacement under the same mechanical load were arranged as follows: HSMs group > LSMs group > THMs group (P < 0.05). The results of dynamic compression test revealed that the peak load of HSMs group could reached 873 N and was 95% of the average yield load of the static compression, and was better than that in THMs group and LSMs group (P < 0.05). Besides, according to the fatigue life-peak load diagram, the ultimate load of HSMs group was more than twice that of THMs group or LSMs group.

**Conclusions:**

The mechanical strength of H-shaped miniplates was superior to two-hole miniplates and L-shaped miniplates in maintaining spinal canal enlargement and spinal stability, and was more excellent in fatigue stability and ultimate load.

## Introduction

Surgical resection with which reduce or relieve the compression of nerve root or spinal cord was widely accepted as an effective treatment for intraspinal occupying lesions, such as intraspinal tumor, symptomatic spinal stenosis caused by hypertrophic facet joint or bulged disc [1–3]. Given that the space-occupying lesions were surrounded by bony structures, part of posterior column structures should be removed to expose required surgical vision [4–7]. Traditionally, decompression laminectomy has been employed as the standard surgical method to open the spinal canal and expose the spinal cord thoroughly [8–12]. Approaches for removing the posterior structures for decompression include partial or total laminectomy, and has been widely used in clinical practices [8–12]. However, as the scope of clinical application expanded and the follow-up time increased, its shortcomings gradually emerged [13–15]. In the clinical environment, even though laminectomy could provide sufficient surgical exposure for safe removal of space occupying lesions in the spinal canal, there were still some problems. The existing problems include the invasion of hematoma into the spinal canal after surgery, iatrogenic spinal stenosis and nerve root adhesion caused by scar tissue formation and adhesion, iatrogenic spinal instability and spinal deformity caused by the removal of posterior column paravertebral muscle attachment points and bone support. These complications may cause persistent or repeated clinical symptoms after surgery, and additional surgical interventions may be required [16–19].

Given that the understanding of the protection and reconstruction of spinal stability was very different in surgeons, how to maintain the stability of spinal biomechanics has become a hot topic in this field [20, 21]. For the past few years, achieving adequate exposure of the spinal canal, complete resection of the space and release of spinal cord compression, maintaining the integrity of the spinal canal and the stability of the spine, and obtaining anatomical and functional reconstruction have become basic principles for treating intraspinal occupying lesions [22–24]. A more recent addition to the surgical armamentarium has been laminoplasty, which was firstly described by Raimondi et al. in 1976 [25]. With the newest kind of surgery, the lamina-spinous process-ligament-muscle complex (LSPLMC) was reattached as a safe mechanical barrier, and the spinal canal was also reconstructed. The laminoplasty could directly decompress the spinal cord by removing posterior compressing structures, such as hyperplastic ligament flavum and ossification of posterior longitudinal ligament (OPLL), or indirectly decompress the spinal cord by increasing and rebuilding the spinal canal volume, and allowing the spinal cord to migrate dorsally away from the anterior compressing structures, such as intervertebral disc and vertebral body. In addition, the laminoplasty could not only achieve sufficient exposure and fully decompression during the surgery, but also prevent postoperative instability of the spine, in line with the mutual unified principle of decompression and stability [26–28]. Currently, it was the most ideal surgical procedure for intraspinal occupying lesions in theory, except for extensive lesions, severe bone destruction or osteoporosis [27–29].

**Fig. 1:**
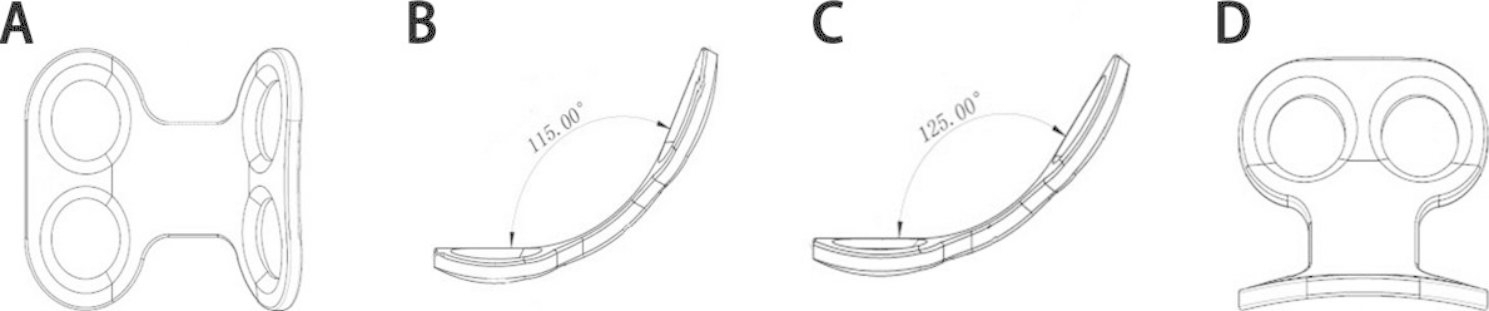
The design of HSMs. (A): The front profile of HSMs. (B): The side profile of HSMs with slope angle of 115°. (C): The side profile of HSMs with slope angle of 125°. (D): The right profile of HSMs

**Fig. 2:**
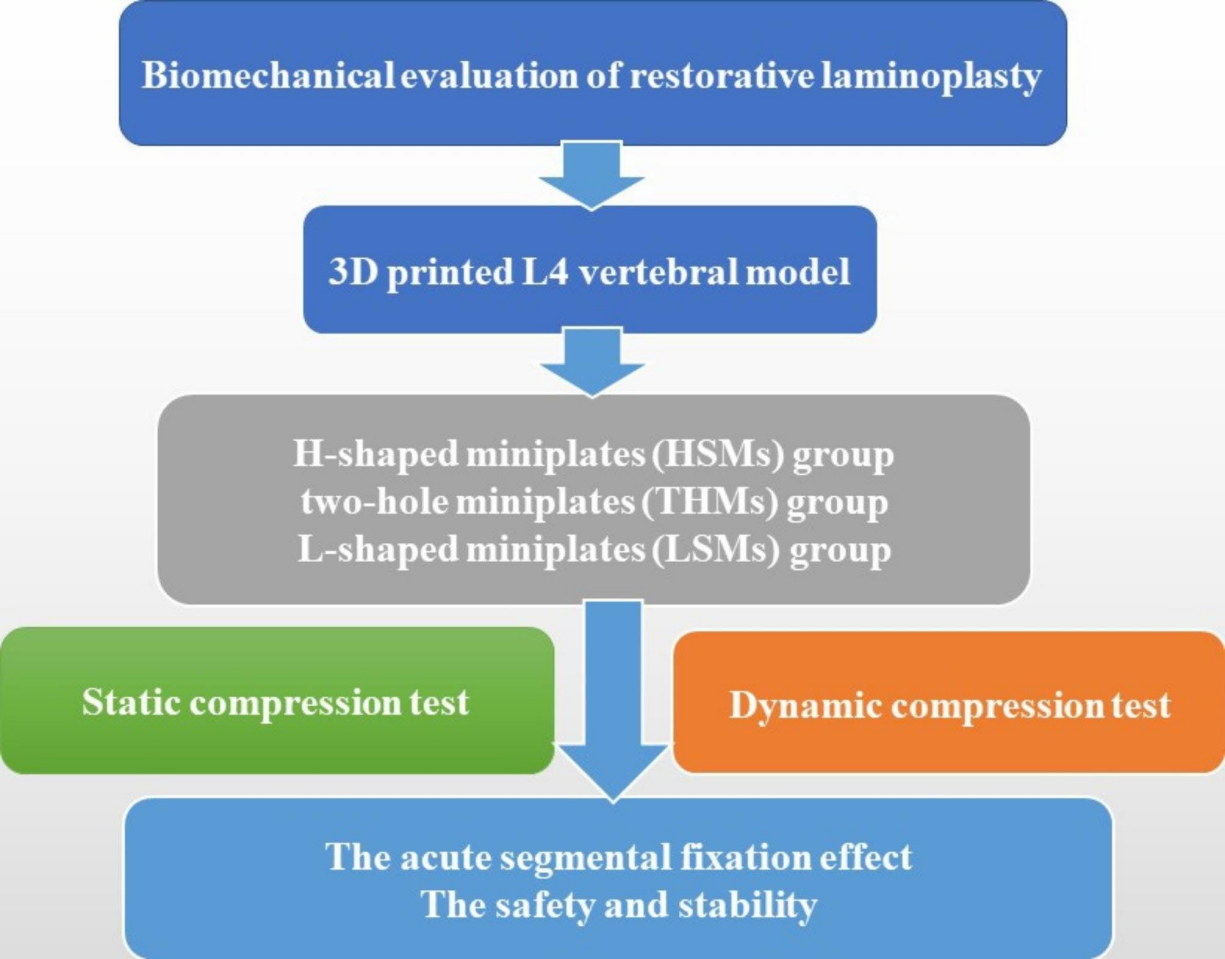
The flow chart of biomechanical experiment

Although surgical methods in which osteotomized laminar complex were repositioned rather than removed have long been performed, they have not been accepted widely yet [3–5, 13, 28]. We believe that one of the reasons may be the lack of proper internal fixation system which matched this technology, and in turn lead to the complicated surgical procedures and unsatisfactory postoperative stability. Thus, the H-shaped miniplates (HSMs) which have been specially designed for restorative laminoplasty by our team were dedicated to the reconstruction of the posterior column of the spine, conformed to the anatomical structure of the lamina, and obtained immediate stability. The specific design parameters of HSMs were shown in Fig. 1. In this study, the biomechanical property of HSMs was tested and compared with that of existing two-hole miniplates (THMs), and L-shaped miniplates (LSMs). The detailed steps were shown in the flow chart (shown in Fig. 2).

## Materials and methods

### Materials preparation

The normal human L4 vertebral model was printed by 3D printing technology with the German EOS Formiga P110 powder sintering system. The materials of L4 vertebral model were PA2200 nylon, which was a kind of special molding material developed by EOS for SLS rapid prototyping equipment.

In PA2200 nylon, the tensile modulus of elasticity was 1.65GPa, tensile strength was 45 ± 3 MPa, maximum elongation was 20 ± 5%, Rockwell hardness could reach 87 HRB, and the printing accuracy could reach 0.1 mm. Meanwhile, in normal human bone, the tensile modulus of elasticity was 0.1-12GPa and the tensile strength was 44 MPa. The properties of PA2200 nylon and normal human bone were similar, and the PA2200 nylon could restore the shape and structure of human vertebral to the greatest extent. Additionally, the materials met the standard of ISO 10993-1, and has been certified to reach the food safety level [30–32].

In this study, a total of 15 PA2200 nylon samples were evenly divided into HSMs group, THMs group, and LSMs group (each group had five samples parallel). Then, the biomechanical properties were measured and analyzed among the 3 groups.

**Fig. 3:**
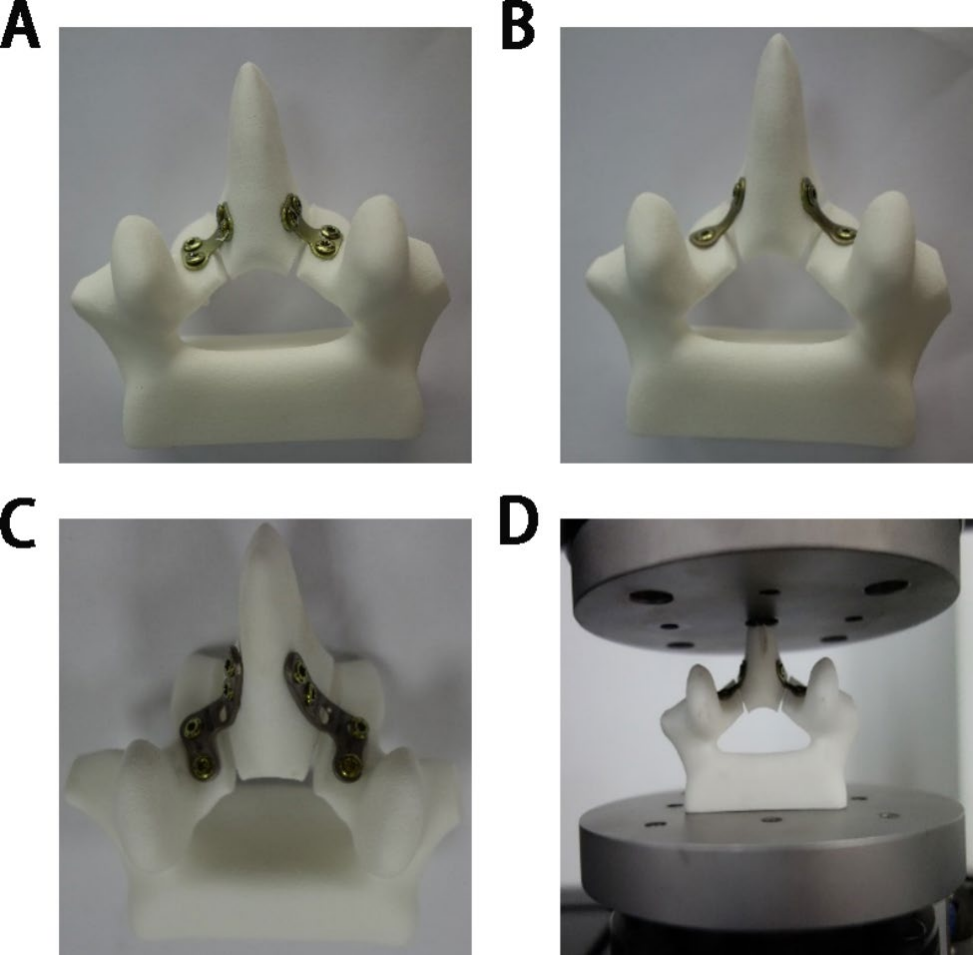
The restorative laminoplasty model of the L4 lumbar spine. (A): Restorative laminoplasty model with HSMs and screws. (B): Restorative laminoplasty model with THMs and screws. (C): Restorative laminoplasty model with LSMs and screws. (D): The experimental schemes of restorative laminoplasty. Five parallel samples were set up for each group

### The restorative laminoplasty model

The laminectomy surgical procedure was performed on the 3D printed L4 lamina model. The spinous process–lamina complex was reattached on the spinal canal, and was fixed by screws (Titanium miniplate system. 2.0*8 mm, China) and HSMs (Titanium miniplate system. 0.6 mm thickness, China) (shown in Fig. 3A), THMs (Titanium miniplate system. 0.6 mm thickness, China) (shown in Fig. 3B), LSMs (Titanium miniplate system. 1.0 mm thickness, China) (shown in Fig. 3C).

The three types of titanium miniplates need to be pre-shaped during the surgical procedure of restorative laminoplasty. However, the pre-shaped range of THMs and LSMs were large, while HSMs were small. Then, the assembled restorative laminoplasty models were subjected to static test and dynamic test respectively.

### Compression test

The assembled restorative laminoplasty models were installed and placed in the middle of the sensor to ensure that the force point was consistent with the loading point (shown in Fig. 3D).

#### Static compression test

The static compression process adopted the speed control mode, and the load was applied at a rate of 5 mm/min till the failure of the model, or the break of the miniplates. The testing machine could collect the load data and the displacement data in real time, and monitor the load-displacement changes.

#### Dynamic compression test

The dynamic compression process adopted the load control mode, with the loading waveform of sine wave, the loading frequency of 5 Hz, the load ratio of 10, and the cycle limit of 5,000,000 times. In order to find out the ultimate load of dynamic compression, the dynamic load was tested with 75% of the average yield load of the static compression. If the cycle number of this stage was less than 5,000,000 times, then the load of the next stage should be decreased by 5-10%; if the cycle number reached 5,000,000 times, then the load of the next stage should be increased by 5% [33–35].

### Statistical analysis

Data are presented as mean ± standard error of the mean (SEM). Differences in mean values of variables of the experimental parameters between groups were assessed by analysis of variance (ANOVA). Post hoc testing of differences between groups was performed by using Duncan’s test when the ANOVA was significant. A p-value < 0.05 was considered to be significant. No adjustment for multiple testing was applied since the statistical analysis was performed in an exploratory manner. Statistical analyses were performed with the SPSS v17.0 software package (SPSS Inc, Chicago, IL, USA).

## Results

**Fig. 4:**
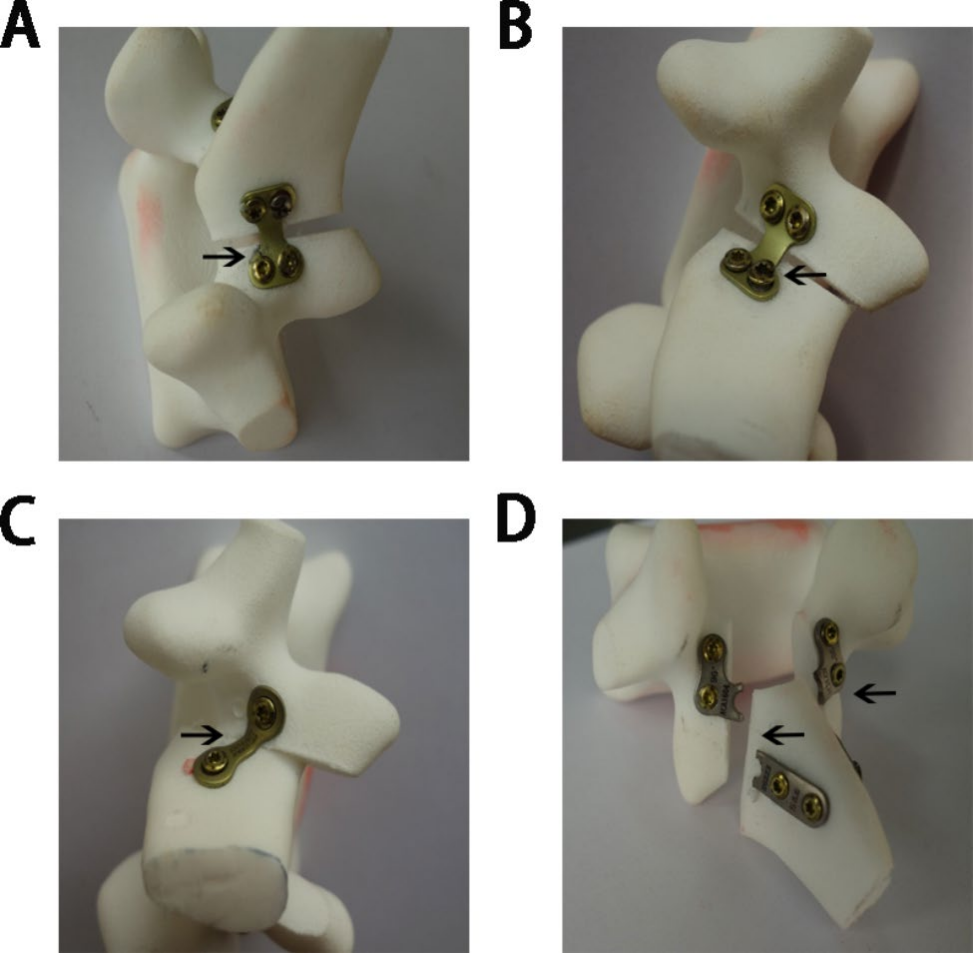
The failure results in static compression test. (A): Plate crack around a screw in restorative laminoplasty model of HSMs. (B): Looseness of a screw tail cap in restorative laminoplasty model of HSMs. (C): The “door close” and the collapse of lamina in restorative laminoplasty model of THMs. (D): The collapse of lamina and plate break in restorative laminoplasty model of LSMs

### Failure results in static compression test

In the test of the static compression, the “door close” and the collapse of lamina occurred in LSMs group and THMs group, but not occurred in HSMs group. The failure results of HSMs group were plate crack around a screw, looseness of a screw tail cap, and no morphology changes occurred in other three assembled restorative laminoplasty models (shown in Fig. 4A and B). In all the five assembled restorative laminoplasty models, the failure results of THMs group were collapse of lamina (shown in Fig. 4C), while the LSMs group were plates break (shown in Fig. 4D).

**Fig. 5:**
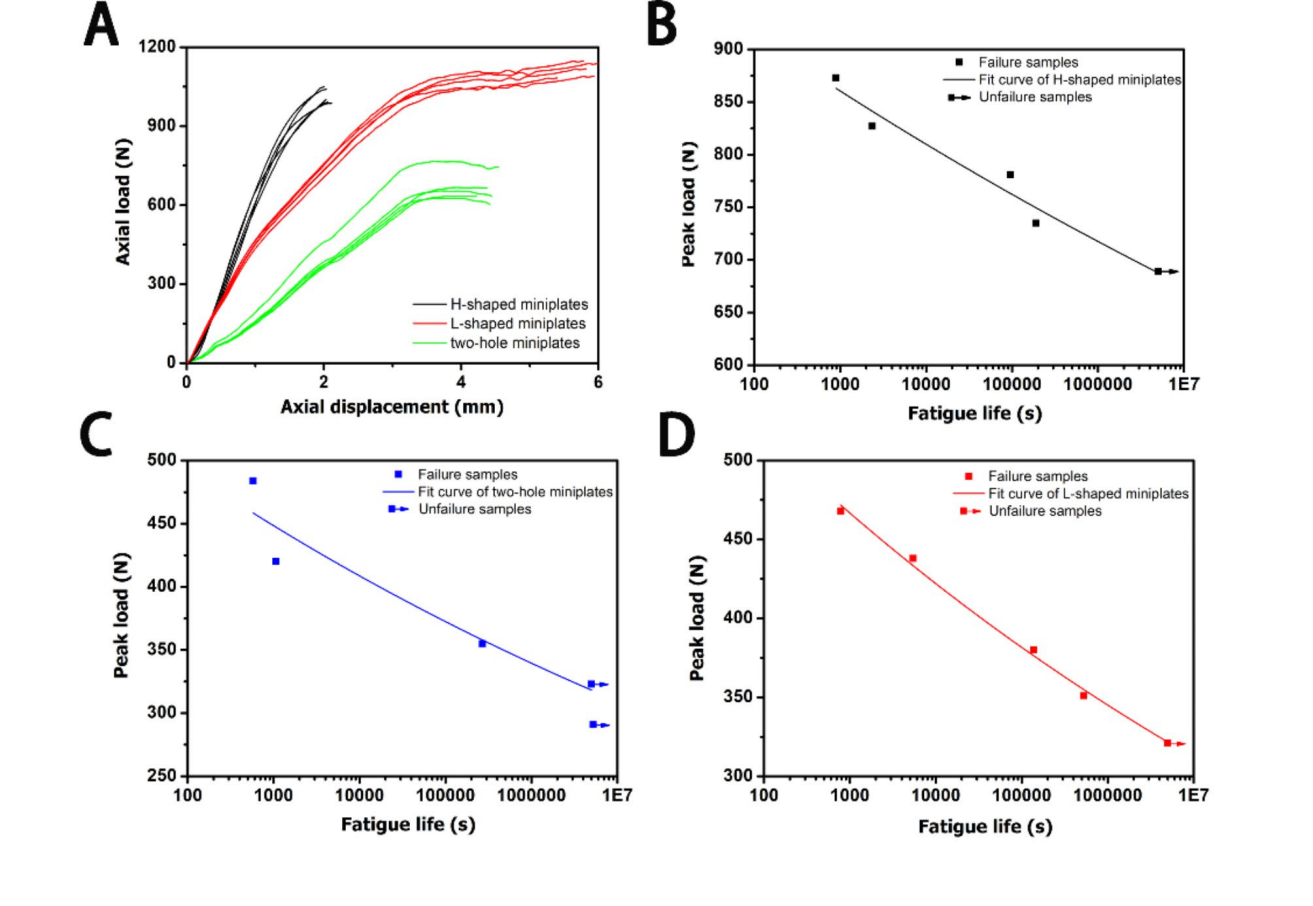
The variation tendency of AD (axial displacement)-AL (axial load) under static compression, and S (fatigue life)-N (peak load) under dynamic compression. (A): The AD -AL results of all miniplates. (B): The S-N results of HSMs group. (C): The S-N results of THMs group. (D): The S-N results of LSMs group. Five parallel samples were set up for each group

### Yield load, yield displacement and compression stiffness in static compression test

**Table 1:** Data of static compression test

Static compression test			
	Yield load, (N)	Yield displacement (mm)	Compression stiffness (N/mm)
HSMs group	919.30 ± 63.66	1.60 ± 0.12	725.23 ± 45.70
THMs group	645.45 ± 18.19^**b**^	3.54 ± 0.13 ^**b**^	207.78 ± 9.19 ^**b**^
LSMs group	584.38 ± 15.02^**b**^	1.52 ± 0.06 ^**d**^	452.11 ± 16.02 ^**b.d**^
**F value** **P value**	61.87 <0.01	562.99 <0.01	413.61 <0.01

Compared with HSMs group: ^a^*P*<0. 05, ^b^*P*<0. 01. Compared with THMs group: ^c^*P*<0. 05, ^d^*P*<0. 01

As was shown in Table 1, The sustainable yield load of HSMs group was greater than that of THMs group and LSMs group (*P* < 0.05), while the sustainable yield load of LSMs group was relatively close to THMs group (*P* > 0.05). No significant difference in yielding-displacement was found between HSMs group and LSMs group (*P* > 0.05), while they were much less than that of THMs group (*P* < 0.05). The compressive stiffness arranged as follows: HSMs group > LSMs group > THMs group (*P* < 0.05).

A diagram of AD-AL (axial displacement-axial load) was drawn based on the data of static test (shown in Fig. 5A). The AD-AL diagram could intuitively reflect the deformability in restorative laminoplasty model of miniplates. It was discovered that the axial displacement gradually increased with the increase of the mechanical load in all three groups (shown in Fig. 5A). Additionally, under the same mechanical load, THMs group showed the largest deformation in the axial displacement, LSMs group followed, and HSMs group had the smallest deformation (shown in Fig. 5A).

### Fatigue endurance in dynamic compression test

**Table 2:** Data of dynamic compression test

Dynamic compression test			
	Peak load (N)	Minimun load (N)	Fatigue life (s)
**LCN of HSMs group** **95%**	873	87	892
**90%**	827	83	2,358
**85%**	781	78	95,360
**80%**	735	74	189,124
**75%**	689	69	5,000,000
**LCN of THMs group**			
**75%**	484	48	577
**65%**	420	42	1,062
**55%**	355	36	268,980
**50%**	323	32	5,000,000
**45%**	290	29	5,000,000
**LCN of LSMs group**			
**80%**	468	47	782
**75%**	438	44	5,432
**65%**	380	38	137,550
**60%**	351	35	523,650
**55%**	321	32	5,000,000

LCN, Load Classification Number

As was shown in Table 2, the fatigue test showed that the peak load of dynamic compression in HSMs group could reach 873 N and was 95% of the average yield load of the static compression, and that the peak load of dynamic compression in H-shaped miniplates was better than that in THMs group and LSMs group. The peak load of dynamic compression in THMs group and LSMs group were only 484 N and 468 N respectively, and were 75% and 80% of the average yield load of the static compression respectively.

A diagram of S-N (fatigue life-peak load) was drawn based on the data of dynamic test (shown in Fig. 5B and D). The S-N diagram could intuitively reflect the fatigue endurance in all three groups. Meanwhile, it could also predict the maximum fatigue load of 5,000,000 cycles without being destroyed, which was also called the ultimate load. The ultimate load of HSMs group was higher than that of THMs group and LSMs group, while the ultimate load of THMs group was relatively close to LSMs group.

## Discussion

To provide an optimal insertion point for the extensor muscles and to endure the stress of body motion, the instruments used for laminoplasty should be strong enough to hold the lamina in place until solid bone fusion obtained. Until now, absorbable fixation, pedicle screws, laminar screws, miniplates and screws have been commonly used to fixate the lamina in laminoplasty [36–38]. However, mechanical problems associated with absorbable fixation, pedicle screws, and laminar screws have been reported [28]. Absorbable fixation may not be strong enough to hold the lamina tightly, and to meet the biomechanics requirements of spine, especially in lumbar or thoracic spine. Moreover, the incidences of postoperative screws loosening and migration in absorbable fixation were high. Although pedicle screws had a rigid fixation effect, they had a great impact on body motion, and exacerbated the degeneration of adjacent segment. The mechanical strength of laminar screws fixation was sufficient to endure the stress of body motion. However, concerns about low fusion rates in patients with multilevel laminoplasty surgery could be raised. Additionally, it was difficult to apply laminar screws to cervical spine due to the angle of laminae of subaxial spine. Moreover, there was a risk of facet joint violation, and that the laminar screws were not readily applicable to certain circumstances (such as eroded laminae or expansile laminoplasty).

Considerable evidences have been showing that the fixation strength of miniplates and screws was comparable with that of pedicle screws and laminar screws in thoracolumbar fusion surgery [22, 28, 39]. Theoretically, a procedure that was strong enough to stabilize a spine segment would be sufficient to hold the lamina until bony fusion obtained. Besides the theoretical mechanical stability, restorative laminoplasty with miniplates fixation had been demonstrated to be a favorable surgical procedure that was not limited by the patient’s age, the surgical site or the number of impaired segments. Moreover, the advantages of miniplates fixation include ease of use and holding the lamina in the almost original position as before until solid bony or strong fibrous unions obtained. However, the clinical effect of restorative laminoplasty with miniplates fixation was limited due to a lack of appropriate internal fixation system which could match this technology in practical terms [13, 28].

In recent years, some surgeons have applied L-shaped or two-hole titanium miniplates with preliminary shaping system to restorative laminoplasty, and achieved satisfactory results [4, 6, 10, 14, 17, 21, 28, 40]. However, the long-term safety and stability need to be further confirmed. Thus, H-shaped titanium miniplates which have been specially designed for restorative laminoplasty by our team were dedicated to the reconstruction of the posterior column of the spine. The H-shaped titanium miniplates have been used clinically in our hospital, and conformed to the anatomical structure of the lamina, and obtained immediate stability. Similarly, the long-term safety and stability of the H-shaped titanium miniplates needed to be further confirmed. Given that the non-reproducible and unpredictable nature of clinical operations and therapeutic effects, and that the serious complication caused by the “door close” and the collapse of lamina, it’s necessary for us to test the biomechanical properties of miniplates.

The posterior column structures of the spine acted as a tension band in the entire spinal system, and subjected to 24–30% of compressive stress and 21–54% of rotational stress [14]. The movement of the spine was complex, mainly include flexion, extension, bending, and rotation. The stress caused by spinal movement would in turn converted into compressive stress on the posterior column structures, and would have greater impact on the jiggle of broken end of lamina. The jiggle of broken end of lamina due to unstable fixation and its cause of poor bony healing were the main causes of the collapse of lamina and spinal stenosis. The stability and bony healing of the miniplates fixed broken end were important factors to maintain the lamina morphology, and to guarantee the integrality of bony vertebral canal and the recovery of neurologic function. The mechanical strength of miniplates fixation should be sufficient to endure biomechanics requirements of spine and stress induced by spine movement. Thus, it’s necessary for us to evaluate the compressive strength of different miniplates on restorative laminoplasty.

In the present study, the static and dynamic compression tests were analyzed to evaluate the mechanical effects of different internal fixation on the post-laminectomy lumbar spine. The “door close” and the collapse of lamina occurred in THMs group and LSMs group, and plates break occurred in the LSMs group, however, no such phenomenon occurred in HSMs group. This suggested that the H-shaped miniplates was superior to L-shaped and two-hole miniplates in maintaining spinal canal enlargement. Meanwhile, the static compression results also showed that the sustainable yield load of HSMs group was greater than that of THMs group and LSMs group, and that the compressive stiffness and the axial displacement under the same mechanical load were arranged as follows: HSMs group > LSMs group > THMs group. This suggested that the H-shaped miniplates were superior in maintaining the axial stability and compression stiffness of the spine. In the dynamic compression test, the peak load of H-shaped miniplates could reach 873 N and was 95% of the average yield load of the static compression, and was better than that in THMs group and LSMs group. Besides, the dynamic compression results also revealed that the ultimate load of HSMs group was more than twice that of THMs group and LSMs group. This suggested that the H-shaped miniplates were more excellent in fatigue stability and ultimate load.

Given that the height, widths, thickness, and angles of the lamina varied from patient to patient, and that anatomy variation of the lamina was large, the specifications, type and radian of the required miniplates would be very different. Additionally, the L-shaped or two-hole miniplates needed to be repeatedly bent in a wide range and pre-shaped largely, which would cause severe damage to the miniplates, such as fatigue or even fracture. Studies showed that the slope angle of lamina varied from 97.8 ± 3.0° at T9 to 129.0 ± 7.5° at L3 [41]. The slope angle of H-shaped miniplates in our test had an arc design of 115° and 125°, and the length was consistent with the evidence. Normally, the H-shaped miniplates could be directly applied to most people and could be directly used for fixation without manual bending. In some cases, a special miniplate bender with slight damage could be used if the anatomy of the laminas were different from normal population.

The manual shaping and radian of miniplates are very important factors because of the uneven and irregularity appearance of laminae and spinous process. The miniplates after bending and shaping should achieve good adhesion and angle, and the force point and stress distribution after miniplates fixation should be guaranteed to prevent iatrogenic spinal stenosis. Our findings indicated that the force point and stress distribution of H-shaped miniplates fixation were completely different from that of the existing L-shaped miniplates and two-hole miniplates, and that the mechanical strength and stability of H-shaped miniplates fixation were better than those of L-shaped miniplates and two-hole miniplates. The broken end of lamina was unstable after fixation of L-shaped miniplates and two-hole miniplates, and still had jiggle or a large range of motion, which was unfavorable to bone healing, and was prone to local pseudojoint formation. However, 3D prisms or pyramids were formed among the force point in H-shaped miniplates fixation, avoiding rotation and displacement caused by the stress distribution of coplanar and coaxial, as well as the instability caused by rotation and displacement.

Currently, the commonly used in restorative laminoplasty were L-shaped miniplates fixation system (metacarpal miniplates for orthopaedics) or two-hole miniplates fixation system (cranial miniplates for neurosurgery). They were existing products in clinical application and have been widely used, while the H-shaped miniplates fixation system was an entirely new product invented by our team and has not been used widely. The thicknesses of L-shaped and two-hole miniplates were constant, while the objective of this research was to compare the H-shaped miniplates with these existing products. The thickness of the L-shaped miniplates was 1.0 mm, and the H-shaped and two-hole miniplates was 0.6 mm. However, the empirical results showed that the mechanical strength of H-shaped miniplates was superior to L-shaped and two-hole miniplates in maintaining spinal canal enlargement, and was more excellent in fatigue stability and ultimate load. After analyzing the causes, the author suggests that it was the morphological difference which caused the uneven force point and stress distribution, and leaded to the excessive concentration of biomechanical load on a certain part.

## Conclusions

For treatment of the laminectomy, a combination of restoration of the anatomic location plus stable internal fixation was necessary on the restoration of the biomechanical property. This research revealed that the H-shaped miniplates were more excellent in mechanical strength, acute segmental fixation effect, safety and stability when compared to the existing L-shaped and 2-hole miniplates. The mechanical strength of H-shaped miniplates was sufficient to endure the stress of body motion, and would contribute to much better fixation effects. Besides the theoretical mechanical stability, the advantages of H-shaped miniplates include ease of use, firmly fixation, anatomic reduction, and holding the lamina in the original position. The reconstruction in restorative laminoplasty with H-shaped miniplates was almost same as that of the conformation of the posterior complex. Therefore, reattached lamina could be easily located in the almost same position as before. This may have potential benefit of facilitating bony healing by minimizing the gap between the cut edges of a grafted lamina and the host bone.

This experiment also had some disadvantages. Complex biomechanical changes were also associated with complex movements such as flexion, extension, bending, and rotation, because of that different mechanical load distribution occured in different postures. The present study mainly discussed the acute segmental fixation effect, safety and stability of miniplates on the restorative laminoplasty, and our research of this thesis was just under this background. The next step in our research is to focus on the effect of spinal movement, and the stress distribution of miniplates and responsible segment during spinal movement. Although a large amount of literature has reported that osseointegration and soft tissue binding ability of miniplates materials could be enhanced through structural or surface modification. Further finite element analysis, animal and related experiments are necessary to assess the effect of multilevel laminoplasty so as to provide further evidence and guidance for clinical application. Further studies should be needed on the association between the restorative laminoplasty and spinal stability.

## Data Availability

The datasets used and/or analysed during the current study are available from the corresponding author on reasonable request.
